# Refurbishing the plasmodesmal chamber: a role for lipid bodies?

**DOI:** 10.3389/fpls.2014.00040

**Published:** 2014-02-24

**Authors:** Laju K. Paul, Päivi L. H. Rinne, Christiaan van der Schoot

**Affiliations:** Department of Plant and Environmental Sciences, Norwegian University of Life SciencesÅs, Norway

**Keywords:** hemi-fusion, lipid droplet, 1,3-β-glucanase, membrane raft, microdomain, oleosin, shoot apical meristem, SNARE

## Abstract

Lipid bodies (LBs) are universal constituents of both animal and plant cells. They are produced by specialized membrane domains at the tubular endoplasmic reticulum (ER), and consist of a core of neutral lipids and a surrounding monolayer of phospholipid with embedded amphipathic proteins. Although originally regarded as simple depots for lipids, they have recently emerged as organelles that interact with other cellular constituents, exchanging lipids, proteins and signaling molecules, and shuttling them between various intracellular destinations, including the plasmamembrane (PM). Recent data showed that in plants LBs can deliver a subset of 1,3-β-glucanases to the plasmodesmal (PD) channel. We hypothesize that this may represent a more general mechanism, which complements the delivery of glycosylphosphatidylinositol (GPI)-anchored proteins to the PD exterior via the secretory pathway. We propose that LBs may contribute to the maintenance of the PD chamber and the delivery of regulatory molecules as well as proteins destined for transport to adjacent cells. In addition, we speculate that LBs deliver their cargo through interaction with membrane domains in the cytofacial side of the PM.

## INTRODUCTION

Recent progress in isolation procedures and proteomic approaches expanded the protein inventory of a generalized plasmodesma (PD), but despite this the PD-proteome is still largely elusive (Bayer et al., 2006; Fernandez-Calvino et al., 2011; Jo et al., 2011). The effort to understand PD functioning from PD composition is faced with several obstacles.

Firstly, PD differ widely among the different cells, tissues and organs of a plant. The main reason for this diversity is the way higher plants growth and development, how they build their body and allocate functions to various parts. Their entire shoot system is derived from the shoot apical meristem (SAM). Daughter cells, produced in cell lineages at the SAM, remain connected via primary PD that are laid down in cell plates. To maintain the necessary symplasmic unity, adjacent lineages become connected via secondarily formed PD. These two distinct mechanisms of PD initiation define the original composition, architecture and function of so-called primary and secondary PD (Rinne and van der Schoot, 1998; van der Schoot and Rinne, 1999). When cells embark on a path to differentiation and specialization, PD structure and function are altered further in correspondence to their position. Thus, rather than being unit structures, PD reflect the functional states of the interconnected cells.

Secondly, the highly dynamic nature of PD in general, but particularly in meristems and developing tissues, might preclude unambiguous establishment of a PD proteome even in a single tissue system. It might turn out that the PD proteome is inherently contingent, and many proteins that associate with PD might be only temporary constituents and regulators, or simply passers-by.

Thirdly, PD do not function in isolation and their proteome is intimately dependent on the regulation of distinct supply routes that deliver components to the exterior and interior of PD. Thus, understanding PD functioning in addition requires identification of the pathways by which proteins are recruited to the exterior and interior of PD, and the mechanisms by which they cooperatively govern PD dynamics. So far, very little is known about these supply routes and how they are coordinated.

**FIGURE 1:**
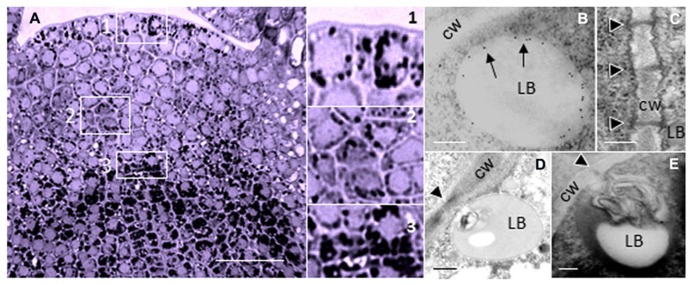
**Lipid bodies in the perennial shoot apex.** **(A)** Shoot apex of hybrid aspen (*Populus tremula × P. tremuloides*) after exposure to short photoperiod results in the accumulation of LBs during initiation of dormancy. LBs are visualized by Sudan Black B. Boxed areas (1–3) are detailed on the right; 1 = Tunica, 2 = Corpus, 3 = Rib meristem/Rib zone. **(B–E)** Electron microscopic images of LBs in shoot meristem of birch (*Betula pubescens*) during chilling-induced release from dormancy. **(B)** Immuno-gold-labeling of 1,3-β-glucanase, peripherally associated with a LB. Arrows point to gold particles that label 1,3-β-glucanase (Form Rinne and van der Schoot, 2004). **(C)** PD in the cell wall of after removal of callosic dormancy sphincter complexes (From Rinne et al., 2001). **(D,E)** Membranous inclusions, probably desmotubule-attached cortical ER in LB that dock PD. Monolayer membranes of LBs are visualized by Osmium tetroxide and tannic acid. Black arrowheads point to PD (From Rinne and van der Schoot, 2004). Bars, **(A)** 50 mm; **(B–E)** 250 nm.

Although PD composition and functioning is most conveniently investigated in the large cells of differentiated tissues, PD functioning is likely to be most versatile and sophisticated in meristematic areas, where morphogenetic signaling is expectably very intense. For several reasons therefore, meristems are of prime interest for the investigation of PD structure and function. Despite their minute size, shoot apices of perennials provide a unique and unexpected experimental opportunity to study PD that cyclically change their structure and function in synchrony with the seasons. Anticipating winter, the SAM of deciduous perennials arrests itself in a morphogenetically deactivated and dormant state. This state is enforced by the production of dormancy sphincter complexes (DSCs). DSCs function as symplasmic circuit breakers that hermetically close all PD by a precise deposition of a callosic mixture around the PD entrance and inside the channel (Rinne et al., 2001). Simultaneously, the isolated cells amass minute lipid bodies (LBs) with a coat of proteins. Associated with the LB surface is a subset of 1,3-β-glucanase (GH17-family) enzymes (**Figure 1**). During chilling-induced release from dormancy these LBs target the plasmamembrane (PM) at, or in close proximity to PD, thereby facilitating restoration of PD functionality (Rinne et al., 2011).

In multicellular organisms LBs have emerged as signaling platforms that deliver proteins and signaling molecules to a variety of intracellular destinations (Murphy, 2012). It seems possible that in plants, LBs have assumed the additional function of a vehicle that delivers proteins to PD for cell-to-cell transport, or regulation and refurbishment of the PD interior (van der Schoot et al., 2011). If so, it would be opportune to analyze the LB proteome and investigate to what degree it overlaps with the PD proteome. In a morphogenetically active SAM, the amount of LBs is far too restricted to make them amenable to biochemical analysis. Fortunately, the dormant apex offers a unique opportunity to isolate sufficient amounts of LBs to analyze their proteome and test this hypothesis. As most of our novel knowledge on LB composition and function is derived from animal systems, we first review crucial findings from the animal literature before we address the question if in plants LBs may contribute to refurbishing the PD interior.

## ORIGIN OF LIPID BODIES

**FIGURE 2:**
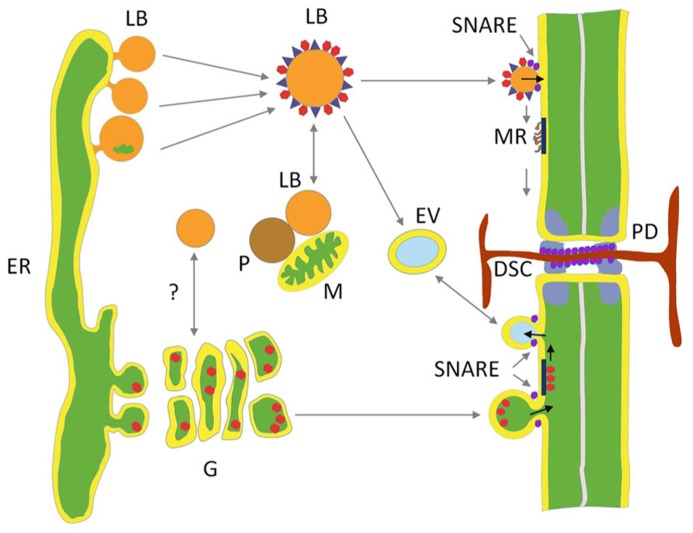
**Hypothetical model depicting two delivery paths to plasmodesmata.** Lipid bodies (LBs) deliver cargo to membrane rafts (MR) at the inner leaflet of the plasma membrane (PM) and the plasmodesmal (PD) channel. LBs are pinched off from specialized areas of tubular ER. Their core of neutral lipids is covered by a protein coat composed of structural proteins, enriched by proteins donated by interacting organelles, like mitochondria (M), peroxisomes (P), early endosome vesicles (EV), and possibly Golgi (G) vesicles. Some of the LBs target PM and transfer proteins to MR that transport them to the PD cavity. In the dormant perennial shoot apical meristem one of the LB proteins is a peripherally associated 1,3-β-glucanase that hydrolyzes the callose plug of the dormancy sphincter complex (DSC). The secretory path delivers cargo that is packed in ER-derived vesicles, among which glycosylphosphatidylinositol (GPI)-anchored 1,3-β-glucanases, that are moved through the Golgi to fuse as exocytic vesicles with the PM, releasing their cargo to the outer side of the PM, where selected GPI-anchored proteins are potentially recruited by MRs for transport to the outer leaflet of the PD neck. Soluble NSF attachment protein receptors (SNAREs) mediate endocytosis, exocytosis, and presumably hemi-fusion of LBs with the PM.

LBs, often called lipid droplets, are of universal occurrence, and have been observed for over a century (Murphy, 2012). In contrast to what the latter name suggests, they are not simple droplets. On the contrary, they are minute membrane-bound organelles, ranging in size from about 0.5 to 2.5 μm, which are produced by specialized areas of the tubular endoplasmic reticulum (ER; **Figure 2**). It is increasingly clear that they are heterogeneous and dynamic entities that serve important regulatory functions.

LBs possess a core of neutral lipids, triglycerides (TAGs) or sterol esters, and a surrounding phospholipid (PL) monolayer (Chapman et al., 2012; Murphy, 2012). This is a relatively stable configuration, with PL acyl-moieties in the hydrophobic core and the charged headgroups in the cytoplasm. TAGs are synthesized in the ER (Huang, 1996) and deposited between the leaflets of the ER membrane. The highly hydrophobic TAGs have low membrane solubility and will “oil out” between the leaflets, forming a lens-like structure (Olofsson et al., 2009). That LBs originate from the ER membrane is supported by their similar PL composition (Fujimoto et al., 2008). However, the detailed mechanism of LB formation has remained elusive, and different models have been proposed (Guo et al., 2009; Walther and Farese, 2009).

Most frequently LB formation is described in terms of a “bulging and budding” model. It depicts LB biogenesis as a process in which nascent LBs bud off from the cytoplasmic side of the ER. As a consequence, the LB monolayer is exclusively derived from the cytoplasmic leaflet of the ER (**Figure 2**). In the “bicelle” or “hatching” model (Ploegh, 2007; Fujimoto et al., 2008), the complete oil lens is cut off from the ER, resulting in a LB monolayer that contains parts of both leaflets. In a third model, the “vesicular budding” model, minute bilayer vesicles are formed that stay tethered to the cytoplasmic side of the ER while a shuttle mechanism transports neutral lipids into its bilayer. This results in a growing LB with a minuscule inner aqueous inclusion that is surrounded by the original luminal leaflet of the ER membrane. This model might imply involvement of coatamers, which assist vesicle formation in the secretory pathway, as knockdown of the COP1/Arf1 machinery interferes with LB formation (Guo et al., 2008). It is possible, however, that all these mechanisms are valid and operate alongside each other. The composition of the protein coat is strongly dependent upon the LB production mechanism, which determines whether only the proteins of the cytoplasmic leaflet of the ER are included or also those at the luminal ER leaflet.

## PROTEIN COMPOSITION OF THE NASCENT LB COAT

The particular structure of a LB restricts what kind of proteins can associate with it. The normal configuration of transmembrane (TM) proteins, with the hydrophilic domains on opposite sides of the membrane, is not feasible in the LB monolayer due to the hydrophobic core. Instead, constitutive LB proteins possess a long hydrophobic domain that forms a hairpin-helix which anchors the protein to the lipid core, while the hydrophilic termini are spread out at the LB surface. Examples of LB proteins with such hairpin topology are caveolin (Martin and Parton, 2006) and the TG- and cholesterol-catalyzing ER enzymes DGAT2 and NSDHL in mammalian cells (Caldas and Herman, 2003; Stone et al., 2006; Murphy et al., 2009), and oleosin and caleosin in plant cells (Lee et al., 1994; Huang, 1996; Chapman et al., 2012; Murphy, 2012).

Alternatively, proteins associate with a LB by embedding amphipathic domains into the monolayer (Brasaemle, 2007; Fujimoto et al., 2008; Guo et al., 2009, Walther and Farese, 2009). Mammalian examples are PAT proteins (including perilipin, adipocyte-differentiation-related-protein [ADRP], and tail interacting protein 47 [TIP47]). They are recruited post-translationally, and are either exclusive for LBs or present in the cytoplasm as well as at LBs. For example, ADRP and perilipin are constitutively associated with LBs via hydrophobic domains, and in absence of neutral lipids they are degraded. In contrast, TIP47 is a soluble cytosolic protein with a terminal hydrophobic domain which is recruited to LBs under elevated fatty acid levels (Wolins et al., 2001), possibly requiring a change in the shape of its hydrophobic pocket (Fujimoto et al., 2008).

An important group of LB-associated proteins are Rab GTPases, which are involved in membrane sorting and targeting (Grosshans et al., 2006; Bartz et al., 2007; Liu et al., 2007). They function as molecular switches which in their active GTP-bound forms recruit effector proteins to mediate vesicle motility, docking, and fusion (Jordens et al., 2005; Liu et al., 2007).

## KISS-AND-RUN ENCOUNTERS AND REFUGEE PROTEINS

Structural studies indicate that LBs interact with other organelles (Binns et al., 2006; Shaw et al., 2008). This is supported by proteomic studies of LBs, revealing the presence of proteins that are characteristic of mitochondria, peroxisomes, endosomes, ER, and PM (**Figure 2**; Goodman, 2008; Guo et al., 2009; Murphy et al., 2009). Transient interactions, mediated by small GTPases, allow the exchange of lipophilic signals and proteins that are embedded in the monolayer or electrostatically attached to its surface (Liu et al., 2008).

That LBs functionally dock to mitochondria (Shaw et al., 2008) is supported for example by fluorescence resonance energy transfer, which provides evidence that their membranes are in direct physical contact (Sturmey et al., 2006; Zehmer et al., 2009). LBs also interact with peroxisomes, to deliver lipids for β–oxidation (Binns et al., 2006; Zehmer et al., 2009) and recruit Rab5 and Rab11 to interact with endosomes (Frolov et al., 2000; Liu et al., 2007). Lipid exchange might proceed in an ATP-independent fashion, as proposed for PM–ER contact sites in yeast (Schnabl et al., 2005), involving transient-inter-compartmental-contact-sites (TICCS; Liu et al., 2007; Zehmer et al., 2009). Alternatively, docking events may be followed by hemi-fusion of the LB monolayer with the outer leaflet of a bilayer structure (Murphy et al., 2009). Cytoplasmic LBs may even usurp ER (Zehmer et al., 2009) as whorls of ER, ribosome-decorated ER, and RNA were detected in the lipid core of some LBs (McGookey and Anderson, 1983; Wan et al., 2007; Zehmer et al., 2009).

Some LBs show Brownian movement, as if waiting for delivery orders, while others move in a coordinated and directional fashion. In animal cells, LBs move on microtubules with dynein motor proteins, but as actin and myosin are also present in the LB proteome they might have ancillary roles (Turró et al., 2006; Bartz et al., 2007; Welte, 2009). In contrast, in plants actin is the major organelle transporter, while microtubules have an assisting role (Collings et al., 2002; Cai and Cresti, 2012). Virtually all encounters between LBs and other organelles are of a transient “kiss-and-run” fashion (van Manen et al., 2005). En route along the cytoskeletal highway, LBs may also pick up proteins and signaling molecules that opportunistically hitch a ride to their destination. Relatively hydrophobic proteins that do not move easily through the aqueous environment of the cytoplasm might piggyback on the lipid shuttle (Welte, 2009). These accidental travelers have been referred to as “refugee proteins” (Hodges and Wu, 2010).

As a direct result of these frequent kiss-and-run encounters and the boarding of opportunistic passengers the LB proteome is surprisingly rich (Welte, 2009; Hodges and Wu, 2010). Proteomic studies of mammalian LBs show that they contain dozens, and perhaps hundreds of proteins (Bartz et al., 2007; Zehmer et al., 2009). For example, a recent investigation identified 125 LB proteins, including Arf1, Arf1 binding protein, coatamers of Arf-1, small G-proteins, lipid synthetic enzymes, chaperones (HSPs), vimentin, calreticulin-3, calnexin, spectrin, heavy-chain myosin, actins, and tubulins (Bartz et al., 2007). As pointed out, the large number of Rabs in these LBs, 18 in total, might indicate that there are distinct classes of LBs with corresponding composition and intracellular destinations (Bartz et al., 2007). Supportive of the validity of such LB inventories is the finding that RNAi screens identified hundreds of genes that are involved in LB biology (Beller et al., 2008; Guo et al., 2008).

Due to the virtual absence of extensive LB-proteome inventories in plants, the number of identified LB-associated proteins is still low. However, there is no a priori reason to expect that the situation in plants is much different from that in animals. The number of peripherally associated LB-proteins, particularly enzymes and signaling molecules, might be equally large. So far, the inventory of proteins found at plant LBs includes among others the structural proteins oleosin and caleosin (Sarmiento et al., 1997; Tzen et al., 1997; Næsted et al., 2000), which both appear to possess enzyme activities (Hanano et al., 2006; Meesapyodsuk and Qiu, 2011; Parthibane et al., 2012), the stress-inducible caleosin RD20 (Aubert et al., 2010), the sterol-dehydrogenase steroleosin (Lin et al., 2002), a peroxygenase (Hanano et al., 2006), a hydroxysteroid dehydrogenase (Li et al., 2007), a lipoxygenase (Hause et al., 2000), an acid lipase (Eastmond, 2004), a patatin-domain lipase (May et al., 2000; Eastmond, 2006), several non-glycosylphosphatidylinositol (GPI)-anchored 1,3-β-glucanases (**Figure 1B**; Rinne et al., 2001, 2011; Rinne and van der Schoot, 2004), the innate immune-response protein calcium-dependent kinase CPK1 (Coca and San Segundo, 2010), glyoxisome receptors (Hause et al., 2000), and various unidentified proteins (Tnani et al., 2011). Many other LB-associated proteins in animal cells have homologs in plants where they may similarly associate with LBs.

## PLANT LBs DELIVER CARGO TO PD

LBs potentially deliver proteins and other associated components to the PD interior in two ways. Firstly, LBs may directly interact with PD and with the cortical ER strands. Transmission electron microscopy showed that during chilling-induced release from dormancy, LB are displaced from random cytoplasmic positions to the PD (Rinne et al., 2001; Rinne and van der Schoot, 2004) where they can usurp membranous material, possibly from ER strands that are continuous with the desmotubule in the center of the PD channel (**Figures 1D,E**). How these LBs deliver the peripherally associated 1,3-β-glucanases (GH17 family proteins) to the callose deposits at the PD channels is uncertain. Secondly, overexpression of eGFP-tagged LB-associated GH17 proteins appeared to target the PM and PD in leaf cells (Rinne et al., 2011). Whereas GPI-anchored eGFP-tagged GH17 proteins labeled PD in punctate patterns, the LB-associated GH17 proteins mostly localized at the PM in distinct sandwich-like patches that are indicative of delivery into some kind of PM domains (Rinne et al., 2011). GPI-anchored proteins are produced in the ER and after post-transcriptional modification send through the Golgi system to the cell’s exterior, where they are anchored to microdomains at the extrafacial leaflets of the PM (see below). It seems possible that, in contrast, LB-associated GH17 proteins and other LB-associated cargo are recruited to membrane rafts (MRs) or microdomains at the cytofacial side of the PM (Rinne et al., 2011). This would require a functional relation or organizational similarity between MRs or microdomains and LBs.

MRs are considered special nano- or microdomains that are composed of sphingoplids, esters and proteins (Simons and Toomre, 2000; Lingwood and Simons, 2010). Interestingly, in adipocytes, the LB-monolayer is covered by unesterified cholesterol (Prattes et al., 2000) and raft-associated signaling proteins like mitogen-activated protein (Yu et al., 1998, 2000) as well as the raft-associated scaffolding protein caveolin-2 (Fujimoto et al., 2001). This prompted Fujimoto et al. (2001) to speculate that LBs function as a novel membrane domain, with caveolin residing in raft-like domains. This “sensational proposal” (van Meer, 2001) warrants a closer look.

## MEMBRANE RAFTS AND DOMAINS

It is well-established that lipid-based rafts in the PM are ordered domains of sterols and highly saturated sphingolipids that arise by self-association within a more disordered environment (Simons and Toomre, 2000; Jacobson et al., 2007). These domains, referred to as lipid rafts (LRs; Simons and Toomre, 2000; Rajendran and Simons, 2005) or MRs (Langlet et al., 2000) were originally conceived in terms of the liquid-ordered (Lo) and liquid disordered (Ld) phases found in purified lipid systems. These model systems did not give a realistic picture of MRs in the PM as they also contain selected TM proteins that are excluded from the Lo phase when reconstituted in a model system (Lingwood and Simons, 2010). Isolation of detergent-insoluble (or resistant) membrane fractions (DIMs or DRMs) yielded a large number of PM proteins that seemed to be part of MRs. In plants such fractions could for example contain leucine-rich-repeat (LRR) as well as other receptor-like kinases (RLKs; Peskan et al., 2000; Shahollari et al., 2004; Lefebvre et al., 2007, 2010) that are implicated in endocytosis and signaling (Duncan et al., 2002; Lingwood and Simons, 2010). However, it appeared that DIMs could not be equated with MRs, and the DIM/DRM-based raft concept has been scrutinized lately (discussed in Tanner et al., 2011). Nonetheless, the existence of PM MRs is not in dispute, and their spatio-dynamic features can be mapped by CSLM, immunochemistry and ultrastructural studies (Berchtold and Walther, 2009; Raffaele et al., 2009; Keinath et al., 2010; Mongrand et al., 2010). The current consensus is that MRs are dynamic nano-scale domains, enriched in cholesterol, sphingolipids and GPI-anchored proteins, which act as membrane-organizing “principles” (Lingwood and Simons, 2010). Nano-sized MRs can be triggered to cluster into larger microdomains by lipid–lipid, protein–protein and lipid–protein interactions (Lingwood and Simons, 2010). Although the PM of plants might differ from that in animal systems in terms of lipid composition, similar organizational principles are likely to apply, with MRs serving comparable regulatory and signaling functions (Mongrand et al., 2010; Jarsch and Ott, 2011; Perraki et al., 2012).

Universally, GPI-anchored proteins are exported via the secretory pathway and segregated into exoplasmic MRs, whereas doubly acylated proteins are recruited by inner leaflet MRs (Simons and Toomre, 2000). The cytofacial MRs are of interest in relation to LBs as these microdomains are thought to function as signaling and docking domains. (**Figure 2**; Mongrand et al., 2010). Recently, remorins a family of plant-specific proteins were identified. Members of one group associate specifically with MRs in a sterol-dependent fashion at the inner PM leaflet, despite their overall hydrophilic nature (Raffaele et al., 2009; Jarsch and Ott, 2011; Perraki et al., 2012). In potato, REMORIN1 (StREM1.3) appears to possess a C-terminal lipid anchor, RemCA, which tethers it into the MRs (Perraki et al., 2012). Remorins are suggested to be scaffolding proteins that participate in the regulation of signaling processes by recruiting PM- and cytoplasmically located proteins into microdomains to preassemble signaling complexes (Jarsch and Ott, 2011). These may include RLKs (Lefebvre et al., 2010). Plant-specific sterols and sphingolipids in MRs can also recruit specific signaling proteins, including RLKs, G-proteins, and stress response- and dynamin-related proteins, as well as 14-3-3 proteins (Stanislas et al., 2009; Mongrand et al., 2010).

## DO LBs CONTAIN RAFT-LIKE DOMAINS?

For mammalian systems the original suggestion of Fujimoto et al. (2001) that LBs may represent a new “membrane domain” seems supported by a number of findings.

For example, the scaffolding protein caveolin-2 of PM rafts can shuttle to LBs in an identical orientation, with its long central hydrophobic helix embedded in the monolayer and both hydrophilic termini in the cytoplasm; significantly it is sequestered in small clusters at the LB monolayer in domains not dissimilar to the MRs in the PM, and it can also shuttle from the ER to LBs as well as to the PM (Das et al., 1999; Ostermeyer et al., 2001, 2004; Brasaemle et al., 2004; Martin and Parton, 2006; Rajendran et al., 2007).

Notably, two PAT family proteins, adipophilin and TIP47, are present at the PM as well as at LBs. Under normal conditions they are dispersed in the PM of macrophages and adipocytes, but stimulation of LB formation by incubation with acetylated low density lipoprotein induces their aggregation in elevated PM domains (Robenek et al., 2009). Although much larger than MRs, roughly 1.0–1.5 μM in diameter, these areas clearly represent membrane domains. That LBs are closely apposed to these elevated PM domains seems remarkable. Cytoplasmically localized TIP47 can associate with LBs by changing its hydrophobic pocket (Fujimoto et al., 2008), and this may also underlie its association with the elevated PM domains. Interestingly, in plants the potato remorin StREM1.3 similarly associates with PM rafts or microdomains by changing the configuration of the short C-terminal anchor RemCA. In the cytoplasm the anchor is unordered but in a non-polar lipid environment it spontaneously folds into a hairpin structure with amphipathic-helices that is inserted into the PM (Perraki et al., 2012). 

In addition, LBs can contain flotillin-1, which is regarded as a true MR marker (Babuke and Tikkanen, 2007). Flotillin-1 and flotillin-2 associate with the MR in the PM through acylation sites (Neumann-Giesen et al., 2004; Morrow and Parton, 2005; Otto and Nichols, 2011), as well as through the prohibition homology domain (PHB) which has a putative hairpin-like topology, similar to that of caveolins (Bauer and Pelkmans, 2006). Flotillin-1 and -2 co-assemble into stable, yet mobile complexes at the PM that act as scaffolds, demarcation sites for targeted cargo delivery (Stuermer, 2011), and signaling platforms (Ludwig et al., 2010). They can also function as sensors that detect changes in membrane tension (Ge et al., 2011) and may guide the budding of MRs to emerging LBs (Neumann-Giesen et al., 2004; Rajendran et al., 2007). Interestingly, flotillins are also present in plants at the PM, and are required for entry of nitrogen-fixing bacteria (Haney and Long, 2010). Similarly, MR-associated remorin of *Medicago truncatula* (MtSYREM1) is specifically induced during root nodulation and it accumulates at rhizobia release sites (Lefebvre et al., 2010) that were earlier characterized by presence of the syntaxin SYP132 (Catalano et al., 2007). Based on this evidence, it is tempting to speculate that in plant cells flotillins and syntaxins (see below) may associate with LBs that align with remorin-decorated MRs.

Interestingly, the oligomeric protein stomatin (Stom), a PM raft-associated integral protein, localizes to the late endosomal compartment, and when overexpressed also to LBs (Umlauf et al., 2004). Live microscopy showed that StomGFP-tagged LBs interact with multiple microtubule-associated vesicles, and that stomatin and caveolin-3 may localize to distinct domains at the LB surface (Umlauf et al., 2004). Stomatin has a topology that enables it to associate with rafts as well as LBs. Its C-terminal domain is necessary for raft formation, whereas the long hydrophobic domain tethers it to LBs (Umlauf et al., 2004), much alike a similar hairpin in caveolin-1 (Bauer and Pelkmans, 2006).

The above examples show that there is a relation between LBs and PM micro domains in both animal and plant systems, although the precise nature of that relation is unclear. In plants, the LB monolayer may not contain cholesterol, and therefore the monolayer might not count as a genuine MR, that is, as LR with associated proteins. This does not preclude interaction or exchange, as proteins could have separate domains for targeting LBs and PM rafts, as in case of stomatin. It seems reasonable to propose that LBs represent some kind of “membrane domain,” the more so, as cholesterol might not always be a prerequisite for domain formation. Recently it was shown that electrostatic protein–lipid interactions can give rise to microdomains independently from cholesterol or lipid phases (van den Bogaart et al., 2011). In any case, the examples lend support to the notion that LBs in some way interact with MRs or microdomains to deliver or exchange proteins and lipids. Interestingly, LBs in animal systems are known to contain a number of soluble NSF attachment protein receptors (SNAREs) that are involved in LB fusion. For example, the SNARE syntaxin5 anchors itself in the lipid core, SNAP23 in the LB monolayer, whereas VAMPP4 associates with the LB surface (Boström et al., 2007; Olofsson et al., 2009; Zehmer et al., 2009). It seems likely that LBs can also undergo hemi-fusion with PL bilayers, such as the PM, permitting the transfer of peripherally associated proteins, such as caveolin (Murphy et al., 2009). Hemi-fusions are in terms of energy expenditure less costly than a bilayer fusion, and easier to perform (Murphy et al., 2009). SNARE- and Rab-assisted transient hemi-fusions between LBs and PM domains could possible explain why some MR proteins can be transferred to LBs and vice versa. For example, interaction of LBs and PM caveolae may allow a transient hemi-fusion for the exchange of the MR protein caveolin (Murphy et al., 2009). Significantly, at the plant PM SNAREs might be distributed in microdomains to mediate exocytosis of secretory vesicles (Sutter et al., 2006), and it is tempting to speculate that LBs might hemi-fuse with the PM at mobile microdomains (**Figure 2**).

## REFURBISHING THE PD INTERIOR: A ROLE FOR LBs?

In general, three pathways could be envisioned through which proteins and other components reach the PD exterior and interior. A pathway that delivers proteins to the PD exterior is the secretory pathway through which GPI-anchored proteins, produced in the ER and modified in the Golgi, reach the cell wall and the PD. GPI-anchored proteins are delivered together with sterols and sphingolipids to the cell exterior, like in animal cells. At the exofacial leaflet of the PM they are anchored to MRs, the assembly of which starts in the Trans Golgi Network (Varma and Mayor, 1998; Lingwood and Simons, 2010). In plants, some of these secreted GPI-anchored proteins are recruited to the exterior of PD. Although these proteins might be released to the outside of the PM in close proximity of PD (Oparka, 2004), they have to move laterally to reach the PD neck (Tilsner et al., 2011). As in animal cells MRs are considered to be relatively mobile platforms, it seems reasonable to assume that in plant cells the MRs can move their resident proteins through lateral displacement to PD. Several recently identified proteins could reach the PD exterior this way. For example, GPI-anchored 1,3-β-glucanases (GH17 family proteins) are exported and transferred to the PD neck, where they hydrolyse callose (Levy et al., 2007; Rinne et al., 2011). The GPI-anchored PD-callose-binding protein (PDCB1), which possesses the carbohydrate binding module family 43 that is also found in a number of GH17 proteins (Rinne et al., 2011), is similarly secreted and transferred to the PD neck to link the PD membrane to the callose deposits in the external sphincter ring (Simpson et al., 2009). Another example is plasmodesmata located protein1a (PDLP1a), one of the eight members of the RLK family PDLP1, which reaches PD via the Brefeldin A-sensitive secretory pathway (Thomas et al., 2008). PDLP1a lacks a GPI domain and instead possesses a 21 amino acid transmembrane domain (TMD) that is necessary and sufficient to target PD (Thomas et al., 2008). The TMD is suggested to contain a sorting signal that interacts with other TM proteins during recruitment into a microdomain (Thomas et al., 2008) and may reach the PD through lateral diffusion in the PM (Tilsner et al., 2011). Several other TMD-containing RLKs, with putative roles in stress response pathways, are also localized to PD (Jo et al., 2011).

**FIGURE 3:**
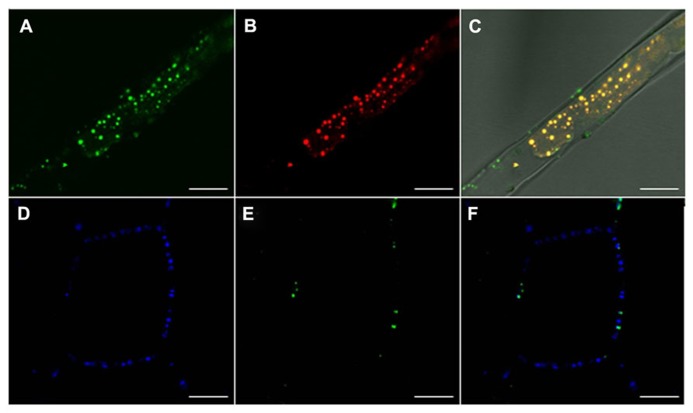
**Lipid body oleosin localizes at the plasma membrane and co-localizes with callose at plasmodesmata.** **(A–C)** Confocal images of *Arabidopsis* root hairs in GFP::Ole2 lines showing **(A)** LB marker-protein, oleosin, GFP::Ole2, **(B)** in connection with lipid after staining with fluorescent dye Nile red. **(C)** Overlay with bright field shows that GFP::Ole2 co-localizes with LBs, except at the plasmamembrane, where GFP::Ole2 containing bodies are free of lipid. **(D–F)** Confocal images of leaf cells of *Arabidopsis *GFP::Ole2 lines showing PD-callose stained with aniline blue **(D)**, and the LB marker-protein GFP::Ole2 **(E)**. **(F)** Overlay shows that GFP::Ole2 has low expression in leaf cells, but co-localizes with callose at PD. Bars, 10 mm.

The other two pathways could deliver proteins to the PD channel, either via the PM or via the ER. Non-secreted proteins could be collected at PD from the cytoplasm via microdomains in the cytofacial leaflet of the PM, either after direct recruitment by scaffolding proteins such as remorin, or after delivery to such scaffold-microdomain clusters by LBs. Delivery of LB cargo is by definition to the PD channel, as the different topologies of the PM double layer and the LB monolayer prevent delivery to the outside of the cell. LB proteins destined for the PD channel could be either permanent residents or only temporary visitors and passers-by to a destination in the adjacent cell. LB routing might be guided by the actin cytoskeleton, as suggested elsewhere (Rinne and van der Schoot, 2004). In case LBs would undergo hemi-fusion with the PM, this would result in lateral diffusion of neutral lipids from the LB core into the PM, and recruitment of cargo to microdomains. Proteins that are peripherally associated with LBs could also peripherally associate with such microdomains, either by embedding amphipatic domains in the cytofacial leaflet or by electrostatic interactions. If so, PM microdomains might shuttle a diverse cargo of LB-delivered non-integral membrane proteins. Most of these proteins might hitch a ride on the LB surface to reach the PD channel for cell-to-cell transport. Alternatively, structural proteins might become embedded in the architectural fabric of the PD channel, a specialized membrane adhesion site (**Figures 2** and **3**; reviewed in Tilsner et al., 2011). Remorin, which accumulates in the PD channel recruits PM- and cytoplasmic proteins into signaling complexes, and there seems no reason why remorin or as yet unidentified scaffolding proteins could not mediate transfer of LB-delivered proteins to the PD chamber. Regardless the precise mechanism, recent investigations showed that LB-associated 1,3-β-glucanase (Rinne et al., 2011) as well as the LB marker oleosin:eGFP target the PM and accumulate at PD (**Figure 3**). This begs the question if oleosin is responsible for targeting LBs to the PM and PD. Oleosin is a structural LB protein that regulates LB size and stability, but which has enzymatic activity and may serve targeting functions. In *Arabidopsis* root hairs, which are devoid of PD except at their base, the transgenic overexpression of oleosin induces LBs that often remain circling in the cytoplasm, but also target the PM. In contrast, in leaf cells they are mostly found at PD, co-localizing with callose (**Figure 3**). Taken together, this suggests that PD are one of the end-destinations of LBs. Oleosin possesses a hydrophobic hairpin that anchors it to the LB core (Huang, 1996; Li et al., 2002). Although oleosin overexpression can induce so-called oleosin-bodies that are unrelated to LBs, it can promote LB formation from the ER in yeast (Jacquier et al., 2013) as well as in *Arabidopsis* root hairs (**Figure 3**). Thus, hairpin-containing plant proteins such as oleosin, and possibly the related LB protein caleosin, can induce LBs in a heterologous system. In line with this, the LB protein steroleosin, which does not have this capacity to induce LBs, is retained in the ER when expressed in protoplasts (De Domenico et al., 2011).

In an alternative route, macromolecular complexes might arrive at the PD channel via strands of ER that terminate at the desmotubule of the PD (Epel, 2009), or via the actin cytoskeleton (Oparka, 2004). Several viruses are known to highjack these systems to reach PD. The desmotubule, centrally located in the PD channel, could also be a potential target of LBs. For example, the protein reticulon which can induce extreme curvature in tubular cortical ER (Tolley et al., 2008) and which could possibly be present at the desmotubule (Tilsner et al., 2011), can associate with LBs (Krahmer et al., 2013). Thus, it seems possible that LBs deliver reticulon from their site of synthesis to the cortical tubular ER, as well as to the interconnected desmotubule.

## PERSPECTIVE

Originally regarded as simple depots for neutral lipids, recent research has revealed that LBs are dynamic organelles that act as transport vehicles, signaling devices, and moving platforms for opportunistic travelers to various destinations, probably including PD. Elucidating LB–PD interactions might facilitate the identification of novel PD components as well as increase understanding of how these components are delivered to the interior of the PD. It could also facilitate the discrimination between structural and modulatory PD components and accidental visitors that are passing through the channel. In the near future, LB isolation, protein purification and sequencing is expected to generate inventories of putative LB-associated proteins. The validity of such inventories will require functional studies to confirm the putative role of LB-associated proteins in the regulation of the PD channel. It is anticipated that such endeavors will reveal that LBs contribute to the functional refurbishment of the PD chamber.

## Conflict of Interest Statement

The authors declare that the research was conducted in the absence of any commercial or financial relationships that could be construed as a potential conflict of interest.
